# Ventilatory Function in Young Adults and Dietary Antioxidant Intake

**DOI:** 10.3390/nu7042879

**Published:** 2015-04-15

**Authors:** Vanessa Garcia-Larsen, Hugo Amigo, Patricia Bustos, Ioannis Bakolis, Roberto J. Rona

**Affiliations:** 1 Respiratory Epidemiology, Occupational Medicine, and Public Health Group, National Heart & Lung Institute, Imperial College London, London, SW3 6LR, UK; 2 Royal Brompton Hospital and Harefield NHS Foundation Trust, Sydney Street, London SW3 6NP, UK; 3 Department of Nutrition, Faculty of Medicine, University of Chile, Independencia 1027, Santiago, Chile; E-Mails: hamigo@med.uchile.cl (H.A.); pbustos@med.uchile.cl (P.B.); 4 MRC-HPA Centre for Environment and Health, Department of Epidemiology and Biostatistics, Imperial College London, London, W2 1NY, UK; E-Mail: i.bakolis@imperial.ac.uk; 5 Department of Psychological Medicine, Weston Education Centre, King’s College, London, SE5 9RJ, UK; E-Mail: roberto.rona@kcl.ac.uk

**Keywords:** antioxidants, flavonoids, FFQ, lung function, young adults, general population

## Abstract

Dietary antioxidants may protect against poor ventilatory function. We assessed the relation between ventilatory function and antioxidant components of diet in young Chileans. Forced expiratory volume in 1 s (FEV_1_), forced vital capacity (FVC), and the ratio FEV_1_/FVC were measured in 1232 adults aged 22–28 years, using a Vitalograph device. Dietary intake was ascertained with a food frequency questionnaire (FFQ) designed for this study, from which nutrient and flavonoid intakes were estimated. Dietary patterns were derived with Principal Component Analysis (PCA). After controlling for potential confounders, dietary intake of total catechins was positively associated with FVC (Regression coefficient (RC) of highest *vs.* lowest quintile of intake 0.07; 95% CI 0.01 to 0.15; *p* per trend 0.006). Total fruit intake was related to FVC (RC of highest *vs.* lowest quintile 0.08; 95% CI 0.003 to 0.15; *p* per trend 0.02). Intake of omega 3 fatty acids was associated with a higher FEV_1_ (RC for highest *vs.* lowest quintile 0.08; 95% CI 0.01 to 0.15 L; *p* per trend 0.02) and with FVC 0.08 (RC in highest *vs.* lowest quintile of intake 0.08, 95% CI 0.001 to 0.16; *p* per trend 0.04). Our results show that fresh fruits, flavonoids, and omega 3 fatty acids may contribute to maintain ventilatory function.

## 1. Introduction

Maintenance of ventilatory function in young adults is important to reduce the risk of its excessive decline later in life [1]. Ventilatory function, measured by forced expiratory volume in one second (FEV_1_) or forced vital capacity (FVC), is a strong predictor of all-cause [2] and cardiovascular mortality [3,4]. Similarly, poor ventilatory function in adults has been related to the development of chronic obstructive pulmonary disease (COPD), which currently represents the third cause of mortality worldwide [5].

Dietary intake might contribute to the preservation of ventilatory function in adults. Observational studies suggest that a higher dietary intake of antioxidants, or a diet rich in fresh vegetables and fruits, is associated with better lung function in the general population [6,7,8,9,10]. Epidemiological studies looking at the effect of the whole diet (dietary patterns) have shown that intake of a diet rich in fruit, vegetable, fish, and wholemeal cereal (referred to by some as a “prudent diet”) is related to a better ventilatory function [11]. Most studies however, have explored either the association with single nutrients, small food groups or dietary patterns, which can limit the scope of interpretation of the effect of diet on lung health.

Chile is a country where the prevalence of chronic respiratory diseases is similar to that observed in more developed nations [12], but that like other South American countries, dietary customs and the availability of food may differ from those of Europe and the United States. To date no evidence has been obtained on the possible association between dietary antioxidants and measures of ventilatory function in adults from Latin America. Investigating the possible protective effect of dietary antioxidants on lung health in young adults might provide evidence on early prevention of rapid ventilatory function decline later in life.

This study investigates the association between ventilatory function (spirometry) and dietary intake of antioxidant nutrients in a representative sample of young adults from Central Chile. We explored this association at nutrient, food and whole diet level. For the latter, using Principal Component Analysis (PCA) we aimed to investigate two established hypothesis in the field of diet and lung function: an *a priori* approach of an association of dietary antioxidants with lung function, as well as a posterior approach in terms of an association of dietary patterns with lung function.

## 2. Methods

### 2.1. Setting and Sample

The study took place in Limache and Olmue, situated in the Central valley of Chile, 141 km from Santiago, with a population of 50,000 inhabitants. Agriculture was the main economic activity in the area at the time of this study, which was carried out between January 2001 and April 2003. The current cross-sectional study is part of a non-concurrent longitudinal study aimed at assessing risk factors for asthma in early childhood and in young adults (22–28 years old) an area where they had preserved prenatal and paediatric records on the growth and development of the children [13].

A sampling frame of 3096 individuals, corresponding to all the births in the Hospital of Limache between 1974 and 1978 was obtained. A simple random sample of 1232 subjects was obtained to investigate poor lung function based on statistical power estimates using the effects of birth weight differences on lung function as outcome. Those who could not be included in the study because of death (3.2%), emigration (11.3%), serving a custodial sentence, disability or lactation (3.3%) and unwillingness to participate (7%) were randomly replaced using the same sampling frame [13].

### 2.2. Ventilatory function

FEV_1_ and FVC were measured in 2001 by trained nurses, using a Vitalograph device model 2120 Spirotrac IV (Buckingham, UK). These measurements were performed following the recommendations of the American Thoracic Society (1987) from which reference values for Chilean adults have been previously derived [14]. FEV_1_ as percentage of predicted value was based on Knudson and colleagues’ recommendations [15]. Any participant who was unable to produce two technically satisfactory manoeuvres after nine attempts was excluded from the study [16]. The highest value for FEV_1_ and FVC produced in up to five satisfactory measures was used in the analyses.

### 2.3. Dietary Exposures

#### 2.3.1. Food Frequency Questionnaire (*FFQ*)

The consumption of 65 food items was assessed in 2001 through the use of a semi-quantitative FFQ designed for this study. The FFQ included a range of staple foods, antioxidant-rich fruits and vegetables, and foods rich in saturated and long chain fatty acids, representing approximately 90% of foods commonly consumed in the Chilean population [17]. The FFQ used in this cross-sectional study was specifically designed to ascertain intake of dietary sources of antioxidants in the Limache cohort and was internally against a repeated 24 h recall questionnaire. The FFQ enquired on average food portion sizes. We report total fruit and vegetable intake, which was obtained from converting the reported portions into daily grams. Data on these foods is reported as daily grams.

None of the participants reported taking nutritional supplements. The fieldworkers who administered the questionnaire were unaware of the antioxidant hypothesis being tested, thus diminishing the risk of information bias.

#### 2.3.2. Nutrient and Antioxidant Estimates

Nutrient estimates were obtained through SofNut software [18] based on The Nutrient Database of the United States Department of Agriculture (USDA), and on the Chilean Chemical Food Composition Table. We previously examined the levels of agreement between the two Tables, and found these were high to excellent for most nutrients, with the exception of polyunsaturated fatty acids (PUFA) were the agreement was moderate [19].

Flavonoid content of foods was estimated using Dutch food composition data [20] as local tables on these compounds were not available. Three major classes of flavonoids were assessed: flavonols, flavones and catechins. Fruits and vegetables rich in antioxidants and vitamins were grouped and their total intake analysed. Total daily intakes of orange, apple, kiwi, strawberry, and mandarin were summed and defined as “total fruit intake”. For “total vegetable intake”, daily total intake (grams) of commonly consumed food items in Chile was considered, namely tomato, potato, pumpkin, carrot, avocado, garlic, onion, beetroot, chard, and sweet pepper.

#### 2.3.3. Assessment of Dietary Patterns

**Figure A1:**
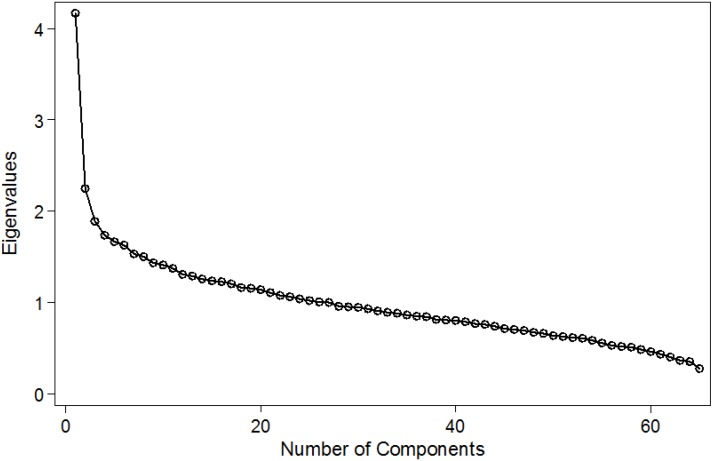
Scree plot of PCA for dietary patterns derived.

Dietary patterns were identified using principal component analysis (PCA). A varimax rotation was applied to improve the interpretability of the patterns obtained. The number of patterns was determined by examination of the scree plot of the eigenvalues. This is a plot of the eigenvalues *vs.* the component number. The aim was to identify an “elbow” which corresponds to the point after which the addition of more components explains relatively little more of the variance. The scree plot from the PCA showed a clear break in the curve after the second component revealing the presence of two dietary patterns (Figure A1 in Appendix). We additionally considered three components but the two first dietary patterns were included in the main analysis as these were the two more clearly differentiated. The PCA was run with all the 65 foods of the FFQ, and a component score was created for each individual for each of the principal components identified. Individual foods that correlated >0.3 or <−0.3 with the varimax rotated dietary patterns (principal components) constituted the dietary pattern (Table A1 in Appendix). Principal component scores were converted into z scores and presented as quintiles to provide a more meaningful interpretation of the results.

### 2.4. Statistical Analyses

Estimates of nutrient intake, BMI and adult weight were expressed in median (inter-quartile range). The rest of the descriptive statistics are presented as means ± standard deviations (SDs).

Maximal FEV_1_ and FVC were the primary outcomes of interest and were analysed as continuous variables. FEV_1_/FVC ratio was also analysed. Food and nutrient/antioxidant variables were analysed as quintile groups in order to make comparisons between highest and lowest levels of intake. With regards to flavonoids, quercetin (the main flavonol) was very highly correlated with total flavonol intake, and therefore flavonols were analysed as a group only. Similarly, as intake of epicatechin was highly correlated with catechins and total catechins (the three main contributors to the intake of total catechins in this study), the analyses are presented as total catechins. Dietary pattern scores and grouped daily (g) intake of fruits and vegetables were also analysed as quintiles.

Multivariable linear regression analysis was used to test associations between ventilatory function outcomes (FEV_1_, FVC and FEV_1_/FVC ratio) and dietary exposures. All models were adjusted for height, sex, age, current smoking, overcrowding, years of fulltime education, socio-economic status, birth weight, body mass index (BMI), and total energy intake (TEI). Socio-economic status in this population was based on indicators appropriate to young adults in Chile as previously reported for this population [21]. Dietary patterns were also adjusted for the effect of each other, as although principal components are uncorrelated, rotations (even orthogonal rotations) can introduce correlations between the patterns. All the dietary exposures were tested for departure from linearity in relation to each of the lung function outcomes with the use of a test for trend. In the current analysis, wheeze or bronchial-hyper-responsiveness (BHR) did not alter the direction of the results observed, and therefore these variables were not included as confounders.

Effect estimates are presented as β-coefficients and 95% confidence intervals. A *p*-value <0.05 was considered to be statistically significant. All analyses were carried out using STATA 12.1 (College Station, Texas, TX 77845, USA). Simes’ procedure was used to explore which associations (if any) remained statistically significant after controlling for multiple comparisons [22].

## 3. Results

Of the 1232 participants, 1187 (96.3%) produced a satisfactory manoeuvre for FEV_1_ and FVC, and had complete FFQ data. The mean BMI was 24.6 kg/m^2^ and 24.7 kg/m^2^ in men and women, respectively. Smoking was common, with over two thirds of males and half of females reporting being current smokers (Table 1).

The average TEI was above the estimated average recommendation (EAR) per day. Carbohydrates were the main source of energy, accounting for nearly 60% of TEI in both men and women (Table 2). Total lipid intake represented a quarter of the TEI. The intake ratio of omega 6 to omega 3 was 33:1. The average intake of total fruits and vegetables was equivalent to 2 and 4 portions per day, respectively. Intake of antioxidant vitamins and minerals was within recommended values. Intake of vitamins and minerals met the daily recommended nutritional intake (RNI) with the exception of selenium that was 82% and 50% of the RNI in men and women, respectively.

The association between outcomes of ventilatory function and intake of flavonoids, antioxidant nutrients and polyunsaturated fatty acids (omega 3 and omega 6) is shown in Table 3. A better FEV_1_ and FVC were positively associated with intake of omega 3 fatty acids (β-coefficient for highest *vs.* lowest quintile of omega 3 intake 0.08 (L); 95% confidence interval 0.005 to 0.15; and β-coefficient 0.08 (L), 95% confidence interval 0.001 to 0.16), respectively. An 80 mL higher FVC was observed in those in the highest *vs.* the lowest quintile of total flavonols, whilst a 70 mL higher FVC was observed in those in the highest quintile of catechin intake compared to the lowest (adjusted *p* per trend 0.006). There was no clear association between the spirometric outcomes studied and antioxidant vitamins or minerals.

Table 4 shows the association between ventilatory function and intake of fruits and vegetables. We found a statistically significantly positive relationship between FVC and total intake of fruits (β-coefficient 0.08; 95% confidence interval 0.003 to 0.15). No associations were observed between spirometric outcomes and total intake of vegetables. After controlling for multiple testing (Simes’ procedure) only the associations between FVC with total fruit intake, and with catechins remained statistically significant.

**Table 1:** General and respiratory characteristics of participants.

Variable	Males (*n* = 542)	Females (*n* = 645)
*Age and anthropometric variables*		
Age (years) Mean (SD)	24.6 (1.4)	24.7 (1.5)
Adult weight (kg) Median (IQR)	70.1 (63.0 to 77.9)	61.5 (54.9 to 70.0)
Adult height (cm) Mean (SD)	168.1 (6.1)	156.3 (5.5)
BMI (kg/m^2^) Median (IQR)	24.7 (22.6 to 27.2)	25.1 (22.6 to 28.6)
Weight at birth (g) Mean (SD)	3184 (488.2)	3192 (508.0)
*Measurements of lung function (Mean (SD))*		
FEV_1_ (L) ^†^	4.12 (0.53)	3.09 (0.38)
FEV_1_ as % of predicted value for age, height and sex	105.2 (10.91)	105.3 (10.69)
FVC (L)	4.78 (0.63)	3.53 (0.44)
FVC as % of predicted value for age, height and sex	104.9 (7.3)	103.2 (8.5)
FEV_1_/FVC	0.86 (0.047)	0.88 (0.048)
Bronchial hyper-responsiveness (BHR) as positive response to methacholine challenge (16 mg/mL) %	6.3	15.5
Wheeze in the last 12 months (%)	26.3	28.4
Ever had asthma (%)	3.1	5.9
Dr diagnosed asthma (%)	2.6	5.4
12 years of full time education (%)	50.0	50.0
*Number of household belongings (as estimation of SES)* (*N* (%))		
0 or 1	75 (13.8)	93 (14.3)
2	164 (30.2)	223 (34.4)
3	173 (31.9)	211 (32.5)
4 or 5	131 (24.1)	122 (18.8)

^†^ Highest of five measurements.

**Table 2:** Daily average dietary intake of foods and antioxidants.

Energy and Nutrients	Males	% TEI	% EAR	% RNI	Females	% TEI	% EAR	% RNI
	Median [IQR]				Median [IQR]			
Total Fruits (g)	182.5 [92.0–210.8]				175.3 [87.4–205.2]			
Total vegetables (g)	432.4 [390.8–462.0]				345.2 [294.8–401.6]			
Total energy intake (TEI) (kcal)	3439 [2665.0–4415.0]		134.9		2279 [1170.0–2844.0]		117.5	
Proteins (g)	129.6 [98.4–163.3]	18.2	292		92.1 [71.0–118.1]	15.4	256	
Carbohydrates (g)	502.6 [385.1–625.2]	58.5	136.0		328.8 [254.4–427.8]	57.7	138.0	
Total lipids (g)	97.9 [71.1–137.6]	23.3	73.1		66.1 [48.2–91.9]	26.9	74.6	
PUFA (g)	26.2 [18.3–36.1]	6.9	106.2		18.7 [13.3–25.5]	7.4	113.8	
MUFA (g)	36.6 [25.5–55.1]	9.6	73.8		23.3 [16.7–35.5]	9.2	70.8	
SFA (g)	26.1 [18.9–38.1]	6.8	61.8		17.1 [11.7–25.1]	6.8	61.8	
Omega 3 (g)	0.24 [0.1–0.5]	0.06	1.2		0.2 [0.1–0.4]	0.08	4.0	
Omega 6 (g)	11.9 [7.7–17.0]	3.1	310		8.5 [5.8–13.1]	3.4	340	
Carotene (µg)	1027.0 [585.6–1707.0]			98	1107.1 [681.2–1876.8]			96
Total vitamin A (µg)	1412.3 [854.4–2121.7]			155	1374.5 [891.7–2328.9]			250
Vitamin C (mg)	133.3 [84.9–235.1]			221	138.7 [81.7–229.2]			230
Vitamin E (mg)	19.6 [14.2–26.3]			131	15.2 [10.9–19.7]			100
Selenium (μg)	163.8 [119.9–203.2]			82	100.6 [77.5–129.7]			50
Zinc (mg)	11.8 [9.2–14.7]			100	9.5 [7.2–10.9]			119
Total catechins (mg)	17.1 [7.8–64.4]			NA	21.2 [9.3–68.5]			NA
Flavonols (mg)	26.2 [15.7–44.3]			NA	24.7 [12.7–41.3]			NA
Flavones (mg)	0.1 [0.04–0.2]			NA	0.1 [0.03–0.2]			NA

% TEI: The percentage of total energy intake; % EAR: the percentage of estimated average recommendation per day; % RNI: percentage of recommended nutritional intake; NA: not applicable.

**Table 3:** The association between components of ventilatory function and estimated daily nutrient and antioxidant intake *.

Nutrient	Quintiles	FEV_1_ (L)	FVC (L)	FEV_1_/FVC
		Difference of Means (95% CI)		
Vitamin C (mg)	1	0	0	0
	2	−0.05 (−0.12 to 0.01)	−0.10 (−0.14 to 0.014)	−0.0002 (−0.01 to 0.01)
	3	−0.002 (−0.07 to 0.07)	0.04 (−0.04 to 0.12)	−0.01 (−0.02 to −0.0005)
	4	0.004 (−0.06 to 0.07)	0.02 (−0.10 to 0.10)	−0.002 (−0.01 to 0.007)
	5	−0.02 (−0.09 to 0.06)	−0.01 (−0.09 to 0.07)	−0.003 (−0.01 to 0.007)
	*p for trend*	0.75	0.47	0.44
Vitamin E (mg)	1	0	0	0
	2	−0.06 (−0.13 to 0.01)	−0.07 (−0.15 to 0.01)	0.002 (−0.006 to 0.01)
	3	−0.004 (−0.08 to 0.07)	0.02 (−0.06 to 0.10)	−0.004 (−0.01 to 0.005)
	4	−0.02 (−0.09 to 0.06)	−0.01 (−0.10 to 0.07)	−0.0002 (−0.01 to 0.01)
	5	0.01 (−0.08 to 0.09)	0.04 (−0.05 to 0.14)	−0.01 (−0.02 to 0.01)
	*p for trend*	0.61	0.23	0.32
Total vitamin A (μg)	1	0	0	0
	2	0.06 (−0.004 to 0.13)	0.05 (−0.03 to 0.13)	0.006 (−0.003 to 0.015)
	3	0.04 (−0.03 to 0.11)	0.06 (−0.02 to 0.14)	−0.004 (−0.013 to 0.004)
	4	0.01 (−0.06 to 0.08)	0.04 (−0.04 to 0.11)	−0.004 (−0.013 to 0.005)
	5	0.02 (−0.06 to 0.09)	0.03 (−0.05 to 0.11)	−0.001 (−0.01 to 0.008)
	*p for trend*	0.78	0.65	0.19
Selenium (μg)	1	0	0	0
	2	−0.01 (−0.08 to 0.06)	0.01 (−0.07 to 0.09)	−0.003 (−0.01 to 0.01)
	3	−0.04 (−0.11 to 0.03)	−0.04 (−0.12 to 0.05)	−0.001 (−0.01 to 0.01)
	4	−0.06 (−0.14 to 0.03)	−0.02 (−0.012 to 0.07	−0.01 (−0.02 to 0.003)
	5	−0.02 (−0.13 to 0.09)	0.06 (−0.06 to 0.18)	−0.01 (−0.03 to 0.0001)
	*p for trend*	0.30	0.90	0.09
Zinc (mg)	1	0	0	0
	2	0.02 (−0.05 to 0.09)	−0.001 (−0.08 to 0.08)	0.01 (−0.003 to 0.01)
	3	−0.05 (−0.12 to 0.03)	−0.04 (−0.12 to 0.04)	−0.002 (−0.01 to 0.01)
	4	−0.01 (−0.09 to 0.07)	0.05 (−0.09 to 0.13)	−0.01 (−0.02 to 0.0001)
	5	0.01 (−0.09 to 0.11)	0.02 (−0.09 to 0.13)	−0.001 (−0.01 to 0.01)
	*p for trend*	0.82	0.48	0.14
Omega 3 fatty acids (mg)	1	0	0	0
	2	0.04 (−0.03 to 0.11)	0.05 (−0.02 to 0.13)	−0.003 (−0.01 to 0.01)
	3	0.05 (−0.02 to 0.11)	0.04 (−0.04 to 0.12)	0.002 (−0.001 to 0.01)
	4	0.08 (0.01 to 0.14)	0.08 (0.003 to 0.16)	0.002 (−0.01 to 0.01)
	5	0.08 (0.005 to 0.15)	0.08 (0.001 to 0.16)	0.001 (−0.01 to 0.01)
	*p for trend*	**0.02**	**0.04**	0.52
Omega 6 fatty acids (mg)	1	0	0	0
	2	−0.08 (−0.14 to −0.01)	−0.08 (−0.15 to 0.001)	−0.002 (−0.1 to 0.01)
	3	−0.03 (−0.10 to 0.04)	−0.28 (−0.11 to 0.05)	−0.003 (−0.01 to 0.01)
	4	−0.01 (−0.08 to 0.10)	−0.001 (−0.08 to 0.08)	−0.002 (−0.01 to 0.007)
	5	−0.005 (−0.08 to 0.07)	0.01 (−0.07 to 0.09)	−0.002 (−0.01 to 0.007)
	*p for trend*	0.51	0.41	0.73
Flavones (mg)	1	0	0	0
	2	0.01 (−0.05 to 0.08)	0.01 (−0.06 to 0.09)	−0.001 (−0.01 to 0.01)
	3	0.02 (−0.05 to 0.09)	0.004 (−0.07 to 0.08)	0.002 (−0.01 to 0.01)
	4	−0.002 (−0.07 to 0.07)	0.003 (−0.07 to 0.08)	−0.002 (−0.01 to 0.01)
	5	0.01 (−0.06 to 0.07)	0.01 (−0.06 to 0.09)	−0.001 (−0.01 to 0.01)
	*p per trend*	0.96	0.85	0.77
Flavonols (mg)	1	0	0	
	2	0.07 (0.003 to 0.14)	0.09 (0.01 to 0.16)	0.001 (−0.01 to 0.01)
	3	0.05 (−0.02 to 0.12)	0.06 (−0.01 to 0.14)	−0.001 (−0.01 to 0.01)
	4	0.05 (−0.02 to 0.12)	0.07 (−0.004 to 0.15)	−0.002 (−0.01 to 0.01)
	5	0.08 (0.01 to 0.16)	0.10 (0.02 to 0.18)	0.002 (−0.01 to 0.01)
	*p for trend*	0.08	0.05	0.99
Total catechins (mg)	1	0	0	0
	2	−0.04 (−0.11 to 0.03)	−0.04 (−0.13 to 0.03)	0.0003 (−0.01 to 0.01)
	3	0.03 (−0.03 to 0.11)	0.06 (−0.02 to 0.13)	−0.01 (−0.02 to 0.003)
	4	0.04 (−0.03 to 0.11)	0.08 (0.003 to 0.16)	−0.01 (−0.02 to 0.002)
	5	0.04 (−0.03 to 0.11)	0.07 (0.01 to 0.15)	−0.01 (−0.01 to 0.004)
	*p for trend*	0.06	**0.006**	0.09

* Multiple linear regression adjusted by age, sex, height, house crowding, educational level, current smoking status, socioeconomic level, birth weight, BMI and TEI.

**Table 4:** The association between ventilatory function and estimated daily intake of fruits and vegetables rich in antioxidants *.

Food Group	Quintiles	FEV_1_ (L)	FVC (L)	FEV_1_/FVC
		Difference of Means (95% CI)		
Total fruits (g)	1	0		0
	2	−0.02 (−0.09 to 0.05)	−0.01 (−0.09 to 0.06)	−0.004 (−0.01 to 0.01)
	3	0.03 (−0.04 to 0.10)	0.04 (−0.04 to 0.11)	−0.002 (−0.01 to 0.004)
	4	0.02 (−0.05 to 0.09)	0.05 (−0.03 to 0.12)	−0.005 (−0.01 to 0.004)
	5	0.05 (−0.02 to 0.12)	0.08 (0.003 to 0.15)	−0.005 (−0.01 to 0.003)
	*p for trend*	0.09	**0.02**	0.23
Total vegetables (g)	1	0	0	0
	2	0.03 (−0.04 to 0.10)	0.02 (−0.06 to 0.09)	0.004 (−0.01 to 0.01)
	3	0.01 (−0.06 to 0.08)	0.05 (−0.03 to 0.13)	−0.01 (−0.02 to 0.002)
	4	0.01 (−0.06 to 0.08)	0.05 (−0.03 to 0.13)	−0.01 (−0.02 to 0.0002)
	5	0.03 (−0.05 to 0.10)	0.05 (−0.04 to 0.13)	−0.002 (−0.01 to 0.007)
	*p for trend*	0.74	0.17	0.10

* Multiple linear regression adjusted by age, sex, height, house crowding, educational level, current smoking status, socioeconomic level, birth weight, body mass index, and TEI.

PCA identified two patterns in these adults, which explained 24.7% of the variance in the original 65 food items (Figure A1 in Appendix). One dietary pattern was highly correlated with animal proteins and starchy food, and the second was characterised by fruits and vegetables (data not shown but available from the authors). The logistic regressions between each pattern and the three respiratory outcomes showed no association (Table 5).

**Table 5:** Association between quintiles of dietary patterns and ventilatory capacity *.

Lung Function	Quintiles of Dietary Patterns	Unadjusted Odds Ratio (SE)	Adjusted Odds Ratio (SE)
FEV_1_	“Animal proteins and starchy foods”		
	1	0	0
	2	0.10 (0.06)	−0.05 (0.04)
	3	0.30 (0.06)	−0.02 (0.04)
	4	0.62 (0.06)	−0.01 (0.04)
	5	0.75 (0.05)	−0.04 (0.05)
	*p for trend*	<0.0001	0.78
	“Fruits and vegetables”		
	1	0	0
	2	−0.0001 (0.06)	0.03 (0.04)
	3	−0.12 (0.06)	−0.03 (0.04)
	4	−0.04 (0.06)	0.03 (0.04)
	5	−0.09 (0.01)	0.03 (0.04)
	*p for trend*	0.11	0.48
FVC	“Animal proteins and starchy foods”		
	1	0	0
	2	0.14 (0.07)	−0.05 (0.04)
	3	0.37 (0.07)	−0.008 (0.04)
	4	0.79 (0.07)	0.01 (0.05)
	5	0.94 (0.07)	−0.03 (0.06)
	*p for trend*	<0.0001	0.93
	“Fruits and vegetables”		
	1	0	0
	2	−0.008 (0.07)	0.03 (0.04)
	3	−0.12 (0.07)	−0.008 (0.85)
	4	−0.01 (0.07)	0.07 (0.04)
	5	−0.10 (0.07)	0.05 (0.04)
	*p for trend*	0.21	0.14
FEV_1_/FVC	“Animal proteins and starchy foods”		
	1	0	0
	2	−0.05 (0.004)	−0.003 (0.004)
	3	−0.008 (0.004)	−0.003 (0.005)
	4	−0.02 (0.004)	−0.005 (0.005)
	5	−0.02 (0.005)	−0.002 (0.007)
	*p for trend*	<0.0001	0.54
Ratio FEV_1_/FVC	“Fruits and vegetables”		
	1	0	0
	2	0.003 (0.004)	0.002 (0.004)
	3	−0.002 (0.004)	−0.004 (0.004)
	4	−0.005 (0.005)	−0.006 (0.005)
	5	0.002 (0.005)	−0.001 (0.005)
	*p for trend*	<0.0001	0.37

* Multiple linear regression adjusted by age, sex, height, house crowding, educational level, current smoking status, socioeconomic level, birth weight, BMI, and TEI.

## 4. Discussion

In this population-based study of young adults, we have found that a better FVC was positively associated with a higher intake of total catechins and of fresh fruits. There was also a positive association between ventilatory function and omega 3 fatty acids but it lost statistical significance after adjustment for multiple comparisons.

Nutrient estimates were obtained from the Chilean Food Composition Table, as an attempt to ascertain local nutrient composition data. Although we used a software based on values from the USDA Food Composition Table, we also used nutrient data calculated from Chilean foods, particularly on fresh vegetables and fruits. Most studies in developing countries rely on larger nutrient datasets from the USA or the UK. We found that level of agreement between the Chilean and British Food Composition Tables were very high [19].

Studies on the relationship between ventilatory function and dietary exposures in Latin American adults (or children) are still lacking. This is a public health challenge as the burden of COPD in these countries appears to be similar to that observed in developed countries [23], and yet, the evidence on environmental risk factors is scant. Observational studies from countries with more advanced economies support our findings of a positive association between lung function and intake of fresh fruit. Strachan *et al.* [10] suggested in the early 90s that a lower intake of fresh fruit was related to a lower lung function (estimated as FEV_1_) in adults, with the effect being observed in both smokers and life-long non-smokers. In a random sample from Scottish adults, Kelly and colleagues [7] found that a higher intake of fresh fruits was related to a higher FEV_1_. A recent case-control study showed that FEV_1_ was statistically significantly lower as the dietary inflammatory index (DII) increased. A higher DII is a reflection of a reduced intake of fresh fruit and vegetables [24].

Prospective studies in adults investigating how fruit intake might be related to lung function show that such an effect would appear to be more evident with changes in consumption of fruits rather than with a regular intake sustained in time [9]. In a seven year follow-up of British adults, Carey and colleagues [9] reported that a reduction in fruit intake was related to a more significant decline of lung function, measured as FEV_1_. A more recent prospective study found that a higher intake of fresh fruit and some of the antioxidants commonly found in these foods would favour a slower decline in lung function (measured as FEV_1_) in adults, the effect being considerable higher in smokers or ex-smokers [25]. Contrary to these findings, Butland and colleagues [8] showed that changes in apple intake were unrelated to FEV_1_ after 10 years of follow-up in adults.

In spite of the growing molecular evidence of the benefits of flavonoids on lung structure [26], epidemiological studies on the association between flavonoids and lung function is still scant. Our results of a positive association between catechins and lung function confirm those reported by Tabak and colleagues [27], who reported a 130 mL higher FEV_1_ in Dutch adults who had the highest *vs.* lowest intakes of catechins. Evidence from molecular studies suggests these flavonoids may have a pivotal role in the pathogenesis of COPD, and to have multiple functions including antioxidant and anti-inflammatory properties [28]. Catechins have been shown to directly stimulate the activity of the nuclear protein Sirtuin 1 (SIRT 1), which regulates a variety of physio-pathological processes in the lung epithelial cells including delaying cellular aging, resistance to oxidative stress, and anti-inflammation [29]. Improving our understanding on the regulation of flavonoid metabolism and its regulation by genes would be a step closer to understanding the mechanisms by which flavonoids are involved in poor lung function and in the aetiology of COPD.

In our study population, the estimated daily intake of fish averaged 10 g per day, which is 30% less than the per capita intake of 5.1 kg/year described for the Chilean population [30]. We found a positive association between FEV_1_ and omega 3 fatty acid intake. We are cautious in the interpretation of this result as after controlling multiple testing, the association was no longer significant. However other observational studies have shown that individuals with a higher intake of omega 6 fatty acids and of isoflavonoids have a reduced risk of COPD [31]. There is evidence that a higher intake of docosahexaenoic acid increases lung function in smokers, but this protective effect is not seen in heavy smokers [32]. This raises the question of whether a higher intake of food sources rich in these compounds could ameliorate or delay the development of COPD by maintaining good lung function.

The use of dietary patterns to assess the relationship between diet and disease has been found on the theoretical conception that foods are not consumed in isolation but as meals, which form a dietary pattern. This observation has prompted several epidemiological studies to look at dietary patterns and respiratory diseases. The evidence on whole diet and lung function is still emerging. McKeever *et al.* [33] found a statistically significant reduction in FEV_1_ in adults eating a “traditional” dietary pattern comprised mainly of potatoes and meat, whilst Shaheen and colleagues [11] found that a “prudent” pattern, comprised of fruit, vegetables, fish and wholemeal cereals, was associated with an improved FEV_1_ in adults.

In contrast to these studies we found no association between measures of ventilatory function and dietary patterns. One possibility for the discrepancy between the PCA analyses is that the patterns of the three studies did not include the same foods, and what researchers choose to define as “prudent” or “Western”, or “traditional” or “animal proteins and starchy food” might imply different food components. It is also possible that dietary patterns from adults in South America may not align with those of European countries, as dietary habits are known to be different between these populations.

The assessment of lung function in our study followed a rigorous standardised protocol, providing reliable data on spirometric values. The response rate was high and participants were representative of the area where the research took place. Whilst FFQs are known to have measurement errors, which usually lead to overestimations of the net dietary intake, the FFQ used in this population was designed *a priori* to take into account the hypothesis we were testing on effect of dietary intake of antioxidants and poor lung function in young adults. Therefore, the list of 65 food items included was mainly concerned with foods with known antioxidant content of vitamins or flavonoids available in this region, as well as foods that are part of the usual diet of Chilean adults.

This is a cross-sectional study with limited ability to infer causality. The dietary intake was self-reported and this might lead to under- or over-reporting of actual intake. In spite of these limitations, the consistency of our findings with recent findings from prospective observational [25] and long term intervention studies [34] showing a protective effect of a higher intake of fresh fruits on adult lung function, would lend support to a possible causal interpretation. We believe that the weak association reported between vegetable intake, omega 3 fatty acids, and lung function outcomes deserves further exploration. We have erred in the side of caution in the analysis because the FFQ has a margin of measurement error and our adjustment for several potential confounders and for multiple comparisons, greatly reduced the chance to find significant associations.

## 5. Conclusions

To our knowledge, this is the first epidemiological study to investigate the association between dietary antioxidants and ventilatory function in adults from Latin America. Our findings suggest that intake of fresh fruits and catechins would contribute to the preservation of lung function in adults and possibly reduce the risk of chronic respiratory diseases later in life.

## Appendix

**Table A1:** Empirically Derived Dietary Patterns. Correlations between food items and dietary patterns (only correlations >0.3 or <−0.3 are included).

Food Item	Dietary Pattern I (Western Diet)	Dietary Pattern II (Fruit and Vegetables)
Orange	0	0
Lemon	0	0.3199218
Kiwi	0	0
Apple	0	0.3203267
Strawberrie	0	0
Mandarin	0	0
Beetroot	0	0.4113356
Chard	0	0.3344303
Sweetpepper	0	0.3768218
Garlic	0	0.3903755
Onion	0	0.4760728
Tomato	0	0.3935958
Potato	0.6293718	0.3563259
Pumpkin	0	0.5209844
Carrot	0	0.4572368
Avocado	0.3387931	0
Bean	0	0.3883224
Lentil	0	0.3531107
Chickpeas	0	0
Meataverage	0	0
Meatnofat	0	0
Meatfat	0.3405617	0
Chickenav	0	0
Chickskin	0	0
Chicknoskin	0	0
Ribs	0.4815942	0
Salmon	0	0
Fish	0	0
Shellfish	0	0
Eggs	0.3899055	0
Bread	0.426953	0
Brownbread	0	0
Knybread	0	0
Pasta	0.4377254	0
Rice	0.3212484	0
Cake	0	0
Oil	0.400673	0
Bacon	0	0
Sausage1	0.3344502	0
Frankfurter	0.4175243	0
Ham	0.5813709	0
Offal	0	0
Margarine	0	0
Butter	0	0
Marbutter	0	0
Marglight	0	0
Mayonaise	0.4619392	0
Maylight	0	0
Wholemilk	0	0
Sskmilk	0	0
Smilk	0	0
Yoghurt	0	0
Cheeseg	0.5296596	0
Maturecheese	0	0
Cottagecheese	0	0
Sugar	0	0
Jam	0	0
Honey	0	0
Coke	0.3835198	0
Juice	0	0
Tealight	0	0
Teadark	0	0
Coffee	0	0
Redwine	0.3425927	0
Salt	0	0
